# Experimental Study of the Seismic Performance of L-Shaped Columns with 500 MPa Steel Bars

**DOI:** 10.1155/2014/105826

**Published:** 2014-05-21

**Authors:** Tiecheng Wang, Xiao Liu, Hailong Zhao

**Affiliations:** ^1^School of Civil Engineering, Tianjin University, Tianjin 300072, China; ^2^Key Laboratory of Coast Civil Structure Safety, the Ministry of Education, Tianjin University, Tianjin 300072, China

## Abstract

Based on tests on six L-shaped RC columns with 500 MPa steel bars, the effect of axial compression ratios and stirrup spacing on failure mode, bearing capacity, displacement, and curvature ductility of the specimens is investigated. Test results show that specimens with lower axial load and large stirrup characteristic value (larger than about 0.35) are better at ductility and seismic performance, while specimens under high axial load or with a small stirrup characteristic value (less than about 0.35) are poorer at ductility; L-shaped columns with 500 MPa steel bars show better bearing capacity and ductility in comparison with specimens with HRB400 steel bars.

## 1. Introduction

Concrete structures with specially shaped columns offer advantages such as avoiding prominent corners in a room, increasing usable floor area, and reducing dead load of the structure combined with the use of light infilled walls, so as to be widely applied in multistoried and high-rise buildings in China. Scholars have conducted great amount of experiment on specially shaped concrete frames and components in recent years [1, 2]. However, specially shaped columns are weak at bearing capacity and seismic performance owing to the relatively smaller cross-section area. The column root is the key part controlling the failure of the total structure according to previous test results of the frames [3, 4]. L-shaped columns are the corner columns in specially shaped concrete structures with asymmetric sections so the internal forced state is even more complex [5].

The use of high-strength steel bars as reinforcement in concrete elements has the potential to reduce problems associated with the placement of reinforcing steel and reduce the costs in shipment. These cost savings are expected to be nearly proportional to the increase in yield stress of the reinforcement. There have been numerous research achievements on high-strength reinforcement in concrete structures recently in the developed countries [6–8]. The required amount of longitudinal reinforcement can be reduced greatly while maintaining almost the same deformability with the specimens using normal-strength bars [9, 10]. In this paper, the application of a new type of steel bar with high strength and good ductility named 500 MPa steel bar is investigated (this kind of steel bar has been recommended by the “Code for design of concrete structures” in China (GB50010-2010)). Although steel bars with strength up to 500 MPa have been widely used in developed countries, such as the United States and European countries, they were not employed in the constructional industry until recently in developing countries like China. Owing to the difference of the building code system between China and developed countries, the former experimental data on 500 MPa steel bars provided by the developed world could only be partially referenced and more physical evidences are needed to guarantee the security and building functions after the utilization of 500 MPa steel in the structures. In order to provide technical reference on employing this material in China and revising the China code “Technical specification for concrete structures with specially shaped columns,” 6 L-shaped column specimens with 500 MPa bars were designed and tested under reverse cyclic loading.

## 2. Experimental Investigation

### 2.1. Test Specimens and Material Properties

**Figure 1:**
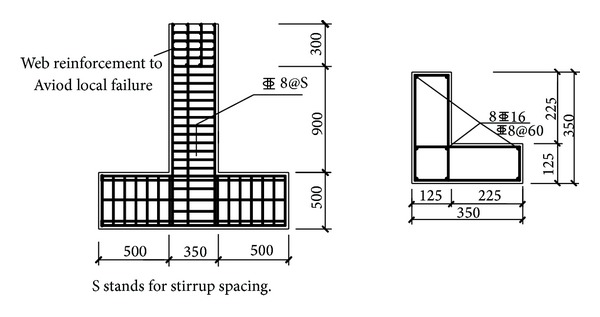
Section dimension and reinforcement details of L-shaped columns.

Six specimens were tested under lateral displacement reversals. The specimens can be assigned into two groups, the first group (L60L, L75L, and L90L) with an axial compression ratio of 0.09 (the corresponding axial load is 200 kN) and the second group (L60S, L75S, and L90S) with an axial compression ratio of 0.24 (the corresponding axial load is 550 kN). The axial compression ratio is defined as *n* = *N*/*f*_*c*_ *A*, where *N* is the axial load, *A* is the cross-sectional area, and *f*_*c*_ is the prism compressive strength of the concrete. These specimens were named by unified rules; that is, the first letter stood for cross-sectional shape, the following number for stirrup spacing, and the last letter for axial compression ratio (L = low axial compression ratio and S = high axial compression ratio).  Figure 1 shows the plan and elevation views of the test specimens. Identical type and amount of longitudinal steel bars without lap splices were applied with a 90° hook at the end to ensure proper anchorage, whereas compound stirrups with 135° hooks were used as transverse reinforcement. The test specimens had 500 mm deep, 1350 mm long, heavily reinforced beams at the bottom, simulating stiff foundation. The bottom beams were fixed to the strong floor by anchor bolts. In addition, one L-shaped column specimen with HPB235 stirrups and HRB400 longitudinal bars, named as L60-400, is selected to be the reference specimen, which has the same geometric property and reinforcement arrangement with the specimen L60L.

C50 grade concrete and 500 MPa reinforcement were used for the specimens. The material properties are shown in Table 1. The specified concrete compressive strength by cubic tests was 51.6 MPa at 28 days, while concrete compressive strength by prism tests was 31.6 MPa. The sizes of the test specimens are 150 mm × 150 mm × 150 mm for cubic compressive strength test and 150 mm × 150 mm × 300 mm for prism compressive strength test. The values from the two kinds of tests are quite different owing to the size effect and the difference in the test equipment. Normal weight aggregate with a nominal maximum size of 25.4 mm was applied for the concrete. The columns were cast upright by a continuous pour from a single batch of concrete.

### 2.2. Test Setup, Instrumentation, and Loading Protocol

**Figure 2:**
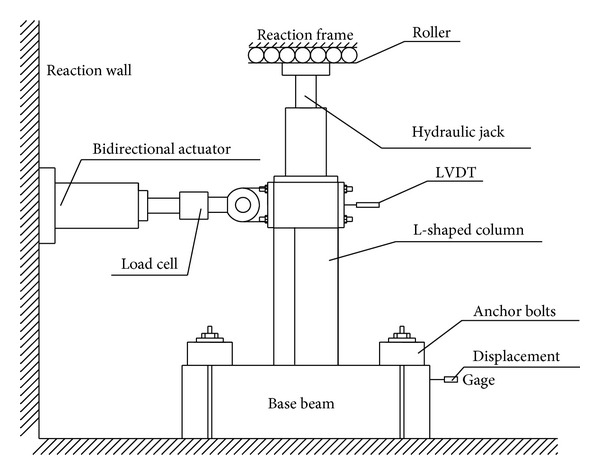
Loading equipment.

**Figure 3:**
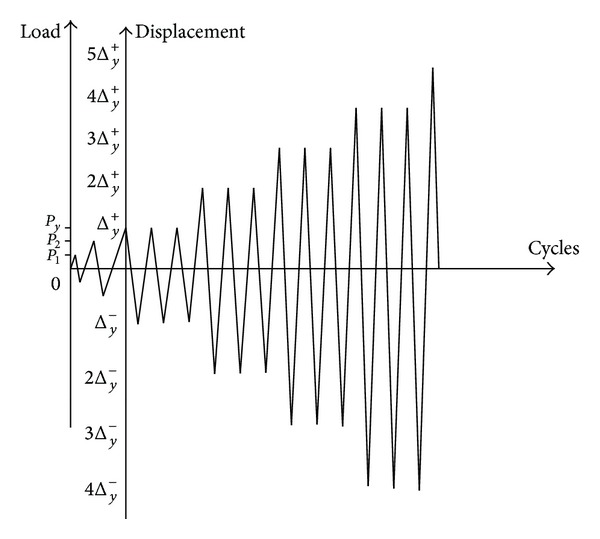
Loading protocol.

The specimens were loaded as shown in Figure 2. Constant axial load was applied at the top of each specimen by a hydraulic jack, whereas lateral load was applied near the top by a bidirectional hydraulic actuator with a 500 kN loading capacity and a ±300 mm linear stroke. The lateral loads and the total column lateral displacements of the column were monitored by a load cell and a linear variable differential transducer (LVDT), respectively. Strains of steel and concrete are monitored by electric resistance strain gauges. On the other hand, two inclinometers were attached to the bottom of the columns to measure the rotation contributed by local discontinuous deformation. The loading protocol is shown in Figure 3. The specimen was defined to fail when lateral load deteriorates to 85% of the peak load or the specimen cannot bear the axial load.

## 3. Test Results and Analysis

### 3.1. Failure Mode and Crack Pattern

The general crack patterns observed for the specimens within the same group were identical. The first flexural horizontal cracks occurred at an applied load of 40 kN for specimens of group one whereas 50 kN for group two, which is located on the web near the bottom of the column. The increase of the applied load resulted in propagation of the cracks and initiation of new flexural cracks along the specimen. A further increase in load extended the existing flexural cracks into flexure-shear cracks and generated vertical cracks along the longitudinal bars. In general, the specimens under higher axial load cracked later and have larger crack angles (angles to horizontal axis).

**Figure 4:**
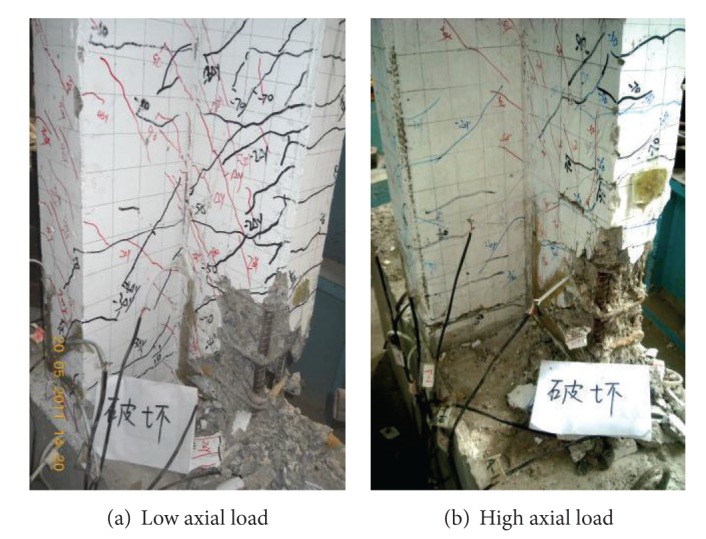
Failure mode of L-shaped columns.

**Figure 5:**
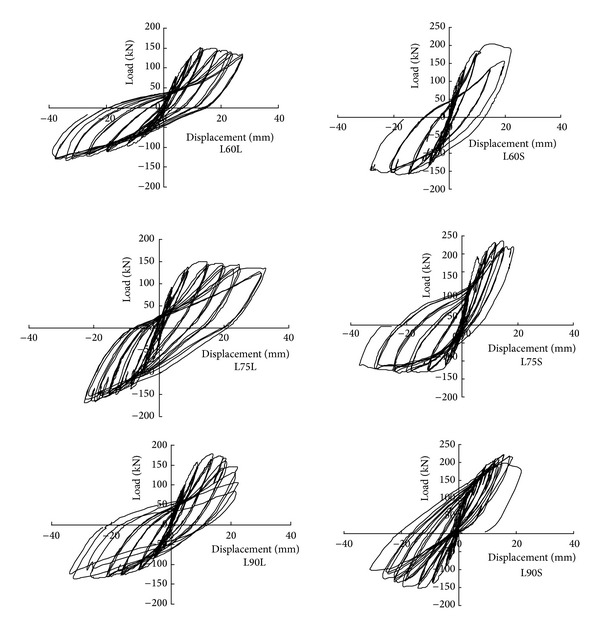
Load-deflection behaviors of L-shaped columns.

There were two kinds of failure modes for these specimens, flexural failure and axial failure. The typical flexural failure was gradual failure which was controlled by crushing of the concrete in the plastic zone near the column base. Specimens that failed in this mode showed obvious postpeak softening behavior and good ductility. In this test, the specimens L60L, L75S, and L75L failed in the flexural mode. The axial failure occurs when severe crushing of the concrete and buckling of the vertical bars in the plastic hinge zone caused a severe degeneration in axial bearing capacity of the specimen and the residual axial bearing capacity is inferior to the applied axial load (Figure 4). This kind of failure is not gradual and there is no evident postpeak softening behavior. In this test, the specimens L60S, L75L, and L75S failed in the axial mode. The plastic hinge was formed in both failure modes for all the specimens. The crack pattern and failure mode are shown in Figure 5. The design shear span ratio for the specimens is 3 and shear effect is not relatively significant, so none of the stirrups yield prior to the failure of steel bars. There were more cracks located on the flanges for specimens with 500 MPa steel bars in comparison with specimens with 400 MPa steel bars, which depicted that the flanges could be more sufficiently employed to resist shear force in specimens with high strength steel.

Measurement results of the strain gauges on stirrups showed that stirrups of L75L, L90L, L75S, and L90S yield in the end. Although stirrups of the other two did not yield in the end, their stress was on high level (the stirrup strain of L60L reaches about 55% of yield strain, and L60S reaches about 73%). The high stresses of stirrups in the plastic zone of the column investigated the fact that stirrups effectively confined the core concrete.

### 3.2. Hysteresis Behaviors

Figure 5 summarizes the lateral load-displacement behaviors of the test specimens.

It is observed that hysteretic loops of specimens with lower axial load have relatively plumper shape, whereas hysteretic loops of specimens with higher axial load have pinched shape. For specimens with identical axial load, hysteretic loops of specimens with less transverse reinforcement covered smaller area. The backbone load-displacement curves of specimens with lower axial load have longer and milder postpeak descending branches, whereas the other specimens have shorter and sharper descending branches. The aforementioned results indicated that increasing transverse reinforcement and decreasing axial load could improve ductility and seismic performance of the specimen.

### 3.3. Bearing Capacity and Displacement Ductility

The displacement ductility is usually measured by displacement ductility factor, expressed as

(1) $$\begin{array}{c} \mu_{\Delta }=\frac{\Delta_{u}}{\Delta_{y}}, \end{array}$$

where *μ*_Δ_ is the displacement ductility factor, Δ_*u*_ is the lateral displacement at the loading point of the column when the specimen fails (specimens are thought to fail when lateral load declines to 85% of the ultimate load), and Δ_*y*_ is the lateral displacement at the loading point when the specimen yields. The calculated results are presented in Table 2.

Analytical results of the first group of specimens (L60L, L75L, and L90L) indicate that the stirrup spacing has no impact on the bearing capacity. The displacement ductility ratio *μ*(L60L) > *μ*(L75L) > *μ*(L90L) indicates that the ductility could be improved by decreasing stirrup spacing. The failing displacements are reduced with the increase in the stirrup spacing due to the better confinement to core concrete provided by the more intense stirrups whereas the yield displacement is independent of it. Similar conclusion on displacement ductility ratio could be obtained for the second group of specimens (L60S, L75S, and L90S).

Specimens under higher axial load have larger bearing capacity, poorer displacement ductility, smaller yield displacements, and failing displacements in comparison with specimens under lower axial load. Concrete of the specimens with higher axial load crush earlier resulted in faster strength degeneration, more brittle member postpeak behavior, and poorer seismic performance.

### 3.4. Specimens with 500** **MPa Bars versus Specimens with HRB400 Bars

Generalized bearing capacity *γ* is defined to measure the bearing capacity eliminating the influence of column height and concrete strength, expressed as

(2) $$\begin{array}{c} \gamma =\frac{M}{f_{c}A}, \end{array}$$

where *M* is the base moment of the column considering second-order effect, *f*_*c*_ is the prism compressive strength of the concrete, and *A* is the cross-section area of the column. The comparison results between L60L and L60-400 are shown in Table 3. The unit of *γ* is “10^−2^ m”.

**Figure 6:**
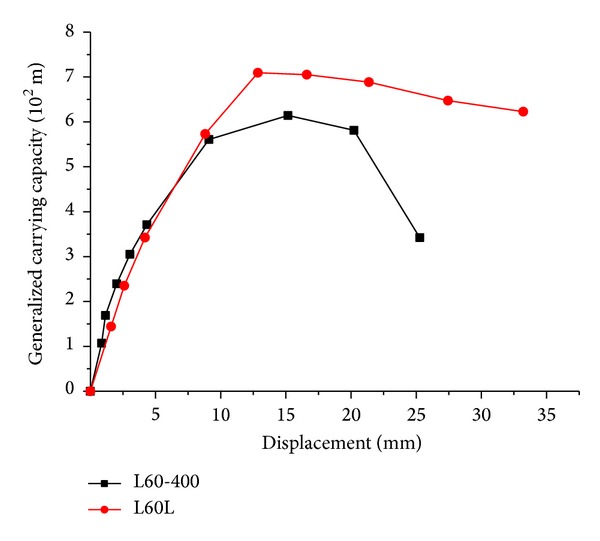
Contrast between L60L and L60-400.

Backbone lateral load-displacement curves of two specimens in the form of generalized bearing capacity are shown in Figure 6.

Table 3 and Figure 6 show that L-shaped columns with 500 MPa bars have bearing capacity which is approximately 15% higher than L-shaped columns with HRB400 bars and larger yield displacement. Their backbone load-displacement curves have longer elastic branch as well as relatively longer and milder descending branch, depicting better seismic performance. However, the initial stiffness of these specimens is decreased.

### 3.5. Curvature Ductility

Curvature ductility is a performance index estimating section ductility which is only related to the rotation of the plastic hinge. The plastic hinge length is a primary parameter in calculating the rotation and curvature ductility factor.

#### 3.5.1. Determination of the Plastic Hinge Length

**Figure 7:**
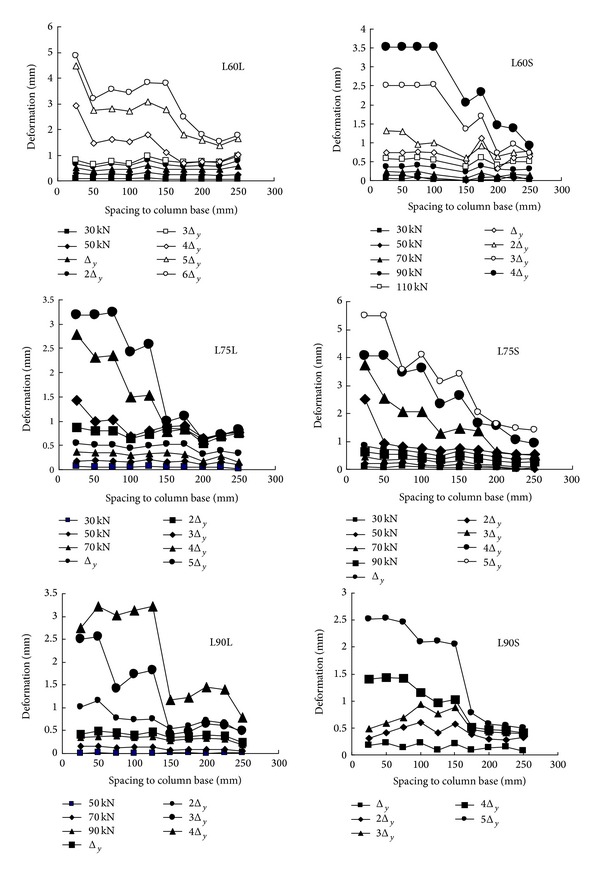
Deformation of border fiber at the bottom of L-shaped columns.

The plastic hinge model is popular in predicting the flexural behavior of the beam or column members due to its simple and clear mechanical concept. In this model, the plastic hinge length *l*_*p*_ is the essential parameter representing the specific length along the member in which the plastic deformation is concentrated, located usually in the end region of the members. In the original work, it is determined by the shape of the curvature profile of the specimen, so it is a mathematical quantity rather than a physical quantity. However, scholars were not stopped from putting their efforts in finding a way to directly measure the plastic hinge length in a test and there have been several methods towards it [11, 12]. The measured plastic hinge length is equal to the mathematical quantity in the original work since they are identical in the definition, that is, the specific length along the member in which the plastic deformation is concentrated. The difference is that the original work got this length through a mathematical way while the measurement method got it by relating it with certain physical quantities such as the quantities concerning the compression strain field. In this test, as a modification of the compression strain field method, we monitored the axial deformation profile along the specimens which is closely related to the magnitude of damage in the critical zone, the concrete spalling/crushing region, and the yielding length of the longitudinal bars as discussed in [11] and found there was a breakpoint in the deformation curves under large displacement stage. The monitored deformation curves of specimens are shown in Figure 7. The breakpoint divided the deformation profile into two parts: one with concentrated deformation and the other without concentrated deformation. We took it for granted that the length of the region with concentrated deformation was just the plastic hinge length *l*_*p*_ based on the aforementioned definition of it. Figure 7 indicates that the plastic hinge length increases with increase of the axial compression ratio.

There are already numerous empirical equations established for calculating the plastic hinge length. These equations are mainly based on regression analysis on test results. Since the data points in this test were too few to establish an equation for calculating *l*_*p*_ of L-shaped columns, we compared the test value of *l*_*p*_ and values obtained from the empirical equations to find out one equation with the best agreement with the test results. This equation will be applied in the future curvature analysis of L-shaped columns temporarily before there is enough experimental data to implement a specific equation for L-shaped columns.  Table 4 shows the contrast between test and calculated values of the plastic hinge length. The formulae used in this paper are described as follows.

(*1) Park and Priestley Equation*. Based on regression analysis on the test results of 20 specimens, Park and Priestley proposed an empirical equation to determine the plastic hinge length, considering the impact of the column height and diameter of the longitudinal bars [13], expressed as

(3) $$\begin{array}{c} l_{p}=0.08L+6d_{s}, \end{array}$$

where *l*_*p*_ is the plastic hinge length, *L* is the column height, and *d*_*s*_ is the diameter of the longitudinal bars.

(*2) Paulay and Priestley Equation.* Paulay and Priestley modified (3) so that the following equation was proposed, in which the impact of different longitudinal bar strength is considered [14]:

(4) $$\begin{array}{c} l_{p}=0.08L+0.022d_{s}f_{y}, \end{array}$$

where *l*_*p*_ is the plastic hinge length, *L* is the column height, *d*_*s*_ is the diameter of longitudinal bars, and *f*_*y*_ is the yield strength of the longitudinal bars.

Equation (4) is the basis for the plastic hinge length equation in the China code “Guidelines for seismic design of highway bridges (JTG/T B02-01-2008)” and the Caltrans code “Seismic Design Criteria.”

(*3) Telemachos Equation.* The equation of the plastic hinge length recommended by Panagiotakos and Fardis [15] for reinforced concrete specimens under cyclic load is

(5) $$\begin{array}{c} l_{p}=0.12L+0.014d_{s}f_{y}, \end{array}$$

where *l*_*p*_ is the plastic hinge length, *L* is the column height, *d*_*s*_ is the diameter of longitudinal bars, and *f*_*y*_ is the yield strength of the longitudinal bars.

(*4) Zahn Equation.* Zahn proposed an equation of the plastic hinge length considering the impact of the axial compression ratio [16]. The plastic hinge length of the specimens is reduced under low axial compression based on test results. Consider

(6) $$\begin{array}{c} l_{p}=\left(0.08L+6d_{s}\right)\left(0.5+1.67n\right), \end{array}$$

where *l*_*p*_ is the plastic hinge length, *L* is the column height, *d*_*s*_ is the diameter of longitudinal bars, and *n* is the axial compression ratio, which will be assigned to 0.3 when *n* ≥ 0.3.

(*5) Equation Recommended by Eurocode 8*. The following equation is recommended by Eurocode 8 [17]:

(7) $$\begin{array}{c} l_{p}=0.1L+0.015d_{s}f_{y}, \end{array}$$

where *l*_*p*_ is the plastic hinge length, *L* is the column height, *d*_*s*_ is the diameter of longitudinal bars, and *f*_*y*_ is the yield strength of the longitudinal bars.

(*6) Equation Recommended by JRA Code.* The following equation is recommended by the JRA code based on the research of Ruangrassamee and Kawashima [18]:

(8) $$\begin{array}{c} l_{p}=0.2L-0.1h, \end{array}$$

where *l*_*p*_ is the plastic hinge length, *L* is the column height, and *h* is the section width along the loading direction, 0.1*h* ≤ *l*_*p*_ ≤ 0.5*h*.

Table 4 shows that values calculated by the Park and Priestley equation, Zahn equation, and equation recommended by the JRA code have a good agreement with the test results. The value calculated by Zahn equation considering the impact of the axial compression ratio has the highest precision, while values calculated by Paulay and Priestley equation, Telemachos equation, and equation recommended by Eurocode 8 code are larger than test values, which will be unsafe in the design procedure.

#### 3.5.2. Curvature Ductility

Based on the research of Priestley and Park [13], the ultimate curvature under the limit state of a cantilever column is located in the plastic zone at the bottom of the column. Equations expressed as (9) could be derived from the curvature profile corresponding to the yield of the specimen:

(9) $$\begin{array}{c} \varphi_{y}=\frac{3\Delta_{y}}{l^{2}}, \end{array}$$

where *φ*_*y*_ is the yield curvature, Δ_*y*_ is the yield displacement, and *l* is the column height.

The ultimate curvature can be calculated by the following equation:

(10) $$\begin{array}{c} \varphi_{u}=\frac{\Delta_{u}-\left(\varphi_{y}l^{2}/3\right)}{l_{p}\left(l-\left(l_{p}/2\right)\right)}+\varphi_{y}, \end{array}$$

where *φ*_*u*_ is the ultimate curvature, Δ_*u*_ is lateral failing displacement of the loading point, and *l*_*p*_ is the plastic hinge length.

The curvature ductility factor of the specimens could be calculated as expressed in

(11) $$\begin{array}{c} \mu_{\phi }=\frac{\varphi_{u}}{\varphi_{y}}. \end{array}$$

The ultimate plastic rotation could be calculated by the following equation:

(12) $$\begin{array}{c} \theta_{p}=\left(\varphi_{u}-\varphi_{y}\right)l_{p}. \end{array}$$

The analytical results from (9)~(12) are presented in Table 5. In the analytical process, the value of *l*_*p*_ measured in the test was used for the specimens except L60-400. For the specimen L60-400, the value of *l*_*p*_ calculated by Zahn equation was used as there is no test value for it and Zahn equation was proved to have the best agreement with the test results in the former section.

The aforementioned test results show that the displacement ductility ratio of the specimen L60L(S) is larger than the specimen L75L(S); however, the curvature analysis gives a reverse result that the curvature ductility factor of the specimen L60L(S) is a little smaller than the specimen L75L(S). These results show that curvature ductility could not continue to be improved by increasing the amount of the transverse reinforcement with a stirrup characteristic value superior to approximately 0.35. The stirrup characteristic value is an index to evaluate the amount of the stirrups, defined as *λ* = *ρ*_*v*_ *f*_*yv*_/*f*_*c*_, where *λ* is the stirrup characteristic value, *ρ*_*v*_ is the volume stirrup ratio, *f*_*yv*_ is the yield strength of the stirrups, and *f*_*c*_ is the prism compression strength of the concrete. The analytical results also show that the ultimate plastic rotation of L60L(S) is 28% (24%) larger than L75L(S), proving that plastic deformability could continue to be improved by increasing the amount of stirrups.

The curvature ductility factor and the ultimate plastic rotation of the specimen L60-400 are smaller than those of L60L and L75L but larger than those of L90L (the stirrup characteristic value of L90L is 0.294 which is greater than 0.267 of L60-400). The poor performance of L90 is perhaps owing to the overlarge spacing. These results indicate that the curvature ductility of the L-shaped columns could be improved utilizing 500 MPa bars under the premise that appropriate stirrup spacing was applied in the design process.

## 4. Conclusion

Based on test results of L-shaped columns with 500 MPa bars under cyclic loading, the following conclusions are investigated.

1. The specimens L60L, L75S, and L75L failed in the flexural mode whereas specimens L60S, L75L, and L75S failed in axial mode in the test; the plastic hinge was formed in both failure modes; the width of the flexural-shear cracks could be limited by increasing the amount of transverse reinforcement; the flanges of L-shaped columns with 500 MPa bars could be employed more sufficiently in comparison with columns with 400 MPa bars.
2. The L-shaped columns with low axial load have better seismic performance and ductility with relatively larger stirrup characteristic value (larger than approximately 0.35), whereas those with high axial load or with too small stirrup characteristic value (less than approximately 0.35) have poorer seismic performance and ductility.
3. The ultimate moment and the displacement ductility factor of the L-shaped columns with 500 MPa steel are 15% and 5% larger, respectively, than the specimen with 400 MPa bars. The yield displacements, the ultimate displacements, and the displacement ductility factors of the L-shaped columns could be improved by increasing transverse reinforcement, while the bearing capacity is not affected by the amount of transverse reinforcement.
4. The plastic hinge length of the L-shaped columns increases with the increase of the axial compression ratio. The values of plastic hinge length calculated by the Zahn equation have the best agreement with the test results.
5. The curvature ductility of L-shaped columns with 500 MPa steel bars is larger than the specimen with 400 MPa bars with stirrup spacing inferior to 75 mm; however, reverse results will be observed with stirrup spacing superior to 75 mm. Curvature ductility could be improved by increasing the amount of stirrups unless the stirrup characteristic value exceeds approximately 0.35. In that case the curvature ductility factors tend to be constant while the ultimate plastic rotations continue to increase.

## Figures and Tables

**Table 1:** Material property of steel bars and concrete of the L-shaped columns.

Specimen	Reinforcement				Concrete		
	Bar	Yield strength/MPa	Ultimate strength/MPa	Elastic modulus/(10^5^ MPa)	Cubic compressive strength/MPa	Prism compressive strength/MPa	Elastic modulus/(10^4^ MPa)
L-shaped column (500 MPa)	500D8	519	800	2.01	51.6	31.6	3.35
	500D16	587	747	2.01			

L60-400	HPB235D8	372.9	454.4	2.13	56.0	37.5	3.90
	HRB400D18	473.5	647.3	2.20			

500D8 and HPB235D8 are, respectively, stirrups of L-shaped columns with 500 MPa bars and with HRB400 bars; 500D16 and HRB400D18 are, respectively, longitudinal bars of L-shaped columns with 500 MPa bars and with HRB400 bars.

**Table 2:** Test results of L-shaped columns.

Specimen	Load/kN		Displacement/mm			Displacement ductility factor
	Yield	Ultimate	Yield	Ultimate	Failure	
L60L	131	150	10.15	12.83	33.20	3.27
L60S	174	204	8.53	14.47	19.58	2.30
L75L	136	150	8.82	13.60	27.05	3.06
L75S	200	235	7.26	14.31	16.27	2.24
L90L	158	177	8.83	13.00	22.01	2.49
L90S	189	220	8.46	15.42	16.78	2.10

Data in the table are test results loading in positive direction; webs of L-shaped columns were under compression at that time.

**Table 3:** Behavior of L-shaped columns with different bars.

Specimen	Generalized carrying capacity *γ*/10^−2^ m		Displacement/mm			Displacement ductility ratio
	Yield	Ultimate	Yield	Ultimate	Failure	
L60L	6.15	7.05	10.15	12.83	33.20	3.27
L60-400	5.24	6.12	8.14	15.24	25.38	3.12

**Table 4:** Comparison between test values and calculated values of length of plastic hinge.

Specimen	Test value/mm	Park and Priestley/mm	Paulay and Priestley/mm	Telemachos/mm	Zahn/mm	Eurocode 8/mm	JRA/mm
L60L	150	180	290.6	257.5	126.1	245.9	170.9
L60S	175	180	290.6	257.5	180	245.9	170.9
L75L	125	180	290.6	257.5	126.1	245.9	170.9
L75S	150	180	290.6	257.5	180	245.9	170.9
L90L	125	180	290.6	257.5	126.1	245.9	170.9
L90S	150	180	290.6	257.5	180	245.9	170.9

**Table 5:** Curvature ductility of L-shaped columns.

Specimen	*l*_*p*_/m	*φ*_*y*_/m^−1^	*φ*_*u*_/m^−1^	*θ*_*p*_/rad	*μ*_*ϕ*_
L60L	0.150	0.04265	0.2860	0.03650	6.71
L60S	0.175	0.03584	0.1371	0.01773	3.83
L75L	0.125	0.03706	0.2651	0.02851	7.15
L75S	0.150	0.03050	0.1256	0.01427	4.12
L90L	0.125	0.03710	0.2020	0.02061	5.44
L90S	0.150	0.03555	0.1234	0.01318	3.47
L60-400	0.130	0.03015	0.1890	0.02065	6.27

Data in the table are test results loading in the positive direction; webs of L-shaped columns were under compression at that time. *l*_*p*_ of specimen L60-400 is calculated by Zahn equation.
